# NETs: the missing link between cell death and systemic autoimmune diseases?

**DOI:** 10.3389/fimmu.2012.00428

**Published:** 2013-01-17

**Authors:** Erika Darrah, Felipe Andrade

**Affiliations:** Division of Rheumatology, Department of Medicine, Johns Hopkins University School of MedicineBaltimore, MD, USA

**Keywords:** cell death, apoptosis, NETs, NETosis, necrosis, autoimmune disease

## Abstract

For almost 20 years, apoptosis and secondary necrosis have been considered the major source of autoantigens and endogenous adjuvants in the pathogenic model of systemic autoimmune diseases. This focus is justified in part because initial evidence in systemic lupus erythematosus (SLE) guided investigators toward the study of apoptosis, but also because other forms of cell death were unknown. To date, it is known that many other forms of cell death occur, and that they vary in their capacity to stimulate as well as inhibit the immune system. Among these, NETosis (an antimicrobial form of death in neutrophils in which nuclear material is extruded from the cell forming extracellular traps), is gaining major interest as a process that may trigger some of the immune features found in SLE, granulomatosis with polyangiitis (formerly Wegener’s granulomatosis) and Felty’s syndrome. Although there have been volumes of very compelling studies published on the role of cell death in autoimmunity, no unifying theory has been adopted nor have any successful therapeutics been developed based on this important pathway. The recent inclusion of NETosis into the pathogenic model of autoimmune diseases certainly adds novel insights into this paradigm, but also reveals a previously unappreciated level of complexity and raises many new questions. This review discusses the role of cell death in systemic autoimmune diseases with a focus on apoptosis and NETosis, highlights the current short comings in our understanding of the vast complexity of cell death, and considers the potential shift in the cell death paradigm in autoimmunity. Understanding this complexity is critical in order to develop tools to clearly define the death pathways that are active in systemic autoimmune diseases, identify drivers of disease propagation, and develop novel therapeutics.

## INTRODUCTION

A hallmark feature of systemic autoimmune diseases is the circulation of autoantibodies that recognize intracellular antigens thought to be expressed by all cells, yet are strikingly associated with specific disease phenotypes and outcomes. These diseases include systemic lupus erythematosus (SLE), rheumatoid arthritis (RA), Sjögren’s syndrome (SS), autoimmune myopathies, systemic vasculitis, and scleroderma. The ubiquitous expression of many autoantigens and their diverse intracellular function and distribution has long posed a problem in understanding the mechanisms by which these proteins become targets of the autoimmune response. It stands to reason that several requirements must be met in order for immune tolerance to be broken to a self-protein: (i) accessibility of the antigen to the immune system; (ii) presence of proinflammatory factors or motifs; and (iii) non-homeostatic state of the protein/altered self (i.e., increased levels of expression or modified protein sequence/structure). Cell death has long been implicated in this process, and for historical reasons, apoptosis has been the most widely studied. However, the recent finding that NETosis can reproduce critical immune features initially ascribed to apoptosis in systemic autoimmune diseases has exposed a previously unrecognized level of complexity in the role of cell death in autoimmunity. Indeed, virtually every form of physiologic cell death that has been described has the potential to meet these requirements, especially in the setting of clearance defects impairing the efficient and rapid removal of dead cells.

Before we lay a framework implicating cell death pathways in the initiation and propagation of systemic autoimmune diseases, it is critical to point out that cell death and damage is a normal and necessary process in a multi-cellular organism. From an evolutionary perspective, no physiologic form of death should have deleterious effects to the host. Any form of cell death, be it organized and planned (i.e., apoptosis) or spectacular and frightening (i.e., NETosis), is physiologic. Redundant mechanisms have evolved to ensure rapid and efficient clearance of dead cell corpses and debris, and development of immune tolerance to self-proteins. Therefore, no form of cell death should have an autoimmune advantage over any other mode of death, unless specific host abnormalities related to unique forms of death exist. The development of autoimmune diseases is known to be highly complex and likely involves the contribution of several genetic and environmental components. Due to this unlikely confluence of predisposing factors, autoimmune diseases are uncommon in the general population with a prevalence of 7.6–9.4% (Cooper et al., 2009). With these caveats in mind, this review will build a framework to better understand the role of cell death in the current pathogenic model of systemic autoimmune diseases with an emphasis on the neutrophil and suggest four theories which integrate NETosis into the existing paradigm.

## THE EXISTING PATHOGENIC MODEL OF CELL DEATH AS A SOURCE OF AUTOANTIGENS IN SYSTEMIC AUTOIMMUNE DISEASES

### A HISTORICAL PERSPECTIVE

The first evidence that suggested a role for cell death in autoimmune disease pathogenesis came from studies by Schwentker and Rivers (1934) in the early 1930s. In these studies, immunization of rabbits with homologous brain suffering autolysis (i.e., spontaneous necrosis) induced anti-brain antibodies and in some cases paralysis, while immunization with freshly isolated brain did not. Although no direct association was made between cell death and the development anti-brain autoantibodies, this observation supported the growing idea that allergic encephalomyelitis and other antibody-mediated diseases may result from autoimmunization against altered tissue generated by the action of pathogens, toxins, chemicals, and/or physical agents (Zoutendyk and Gear, 1951). Later, Burnet (1959) proposed that immune cells reactive with antigenic determinants not readily accessible to the immune system (only in inflamed or traumatized organs) may not be eliminated and be responsible for inducing a vicious cycle of inflammation, most readily when the organism is under stress. This was an important step in recognizing that normally sequestered or altered self-proteins may drive autoimmunity by stimulating autoreactive cells that were not eliminated or rendered tolerogenic during development.

The seminal description of apoptosis as a programmed form of cell death, distinct from necrosis, in 1972, influenced subsequent studies of cell death and ultimately shaped our current pathogenic model of autoimmune diseases (Kerr et al., 1972). It is likely that if another form of programmed cell death had been identified at that time (e.g., necroptosis or NETosis), the current paradigm of cell death and autoimmune disease pathogenesis, may very well center around a different form of death. A central role for apoptosis in autoimmune disease pathogenesis began to emerge in 1990 when two studies provided evidence that apoptosis was a driver of inflammation in autoimmunity. Bell and colleagues observed that nucleosomes released from cells dying by apoptosis could stimulate the production of anti-DNA antibodies *in vitro*, and suggested that this process may occur *in vivo* in SLE (Bell et al., 1990; Bell and Morrison, 1991). Rumore and Steinman (1990) identified that patients with SLE have circulating DNA “closely resembling the characteristic 200 bp ladder found with oligonucleosomal DNA,” and suggested that this DNA may be produced by apoptotic cells. They also suggested the possibility that oligonucleosomal DNA generated during apoptosis may escape phagocytosis, and thus gain access to the extracellular fluid. Later, soluble nucleosomal DNA was found in circulation in other autoimmune diseases including SS, scleroderma, and anti-neutrophil cytoplasm antibodies (ANCA)-associated vasculitis (Holdenrieder et al., 2006), as well as in synovial fluid in RA (Yu et al., 1997).

Although the model linking apoptosis to the pathogenesis of autoimmune diseases was gaining momentum, there was no direct evidence that dead or dying cells were active participants in the process. This culminated in 1994 when two papers were published that placed the apoptotic cell in the spotlight as an important factor in SLE pathogenesis. The first paper, by Emlen et al. (1994), described that patients with SLE have accelerated lymphocyte apoptosis *in vitro* and suggested that “abnormal apoptosis of lymphocytes in SLE may provide a source of extracellular nuclear antigen to drive the immune response and to allow the formation of immune complexes (IC).” Shortly thereafter, a paper by Casciola-Rosen et al. (1994) revealed that SLE autoantigens clustered at the surface of apoptotic blebs (membrane protrusions that form on cells dying by apoptosis). The novelty of this paper was that it showed a model in which not only DNA, but other autoantigens (i.e., ribonucleoproteins, RNP) are potentially exposed to the immune system during apoptosis. Moreover, it proposed that during this process, autoantigens can suffer changes in immunogenicity as result of clustering and potentially through posttranslational modifications.

Based on these studies as well as several others, apoptosis has become a critical part of the pathogenic model of autoimmune diseases, and has been widely considered to be the source of autoantigens (e.g., DNA and RNP) and adjuvants (e.g., HMGB-1) that can initiate and propagate the autoimmune process (Lovgren et al., 2004; Vollmer et al., 2005; Marshak-Rothstein and Rifkin, 2007; Urbonaviciute et al., 2008). However, since apoptotic cells are largely considered anti-inflammatory, secondary necrosis of apoptotic cells is a further step that is necessary in this model to expose the cellular contents of dying cells to the immune system. The extracellular exposure of intracellular antigens and endogenous adjuvants, together with the abnormal clearance and/or response to these molecules is the most widely-accepted hypothesis in the paradigm of autoantibody production and systemic autoimmunity (Suber et al., 2008; Munoz et al., 2010; Mahoney et al., 2011; Wickman et al., 2012).

## THE COMPLEXITY OF CELL DEATH

### THREE MAIN CATEGORIES OF CELL DEATH

Given the wide acceptance of the hypothesis that apoptosis plays a central role in autoimmune disease pathogenesis, it is surprising that no successful therapeutics have been developed which target this pathway in human autoimmune diseases (e.g., to improve the clearance of dying cells). If we accept that cell death is playing an important role, this likely reflects a lack of understanding of the vast complexity of cell death mechanisms, something that is only beginning to be appreciated. According to the last recommendations of the “Nomenclature Committee on Cell Death,” cell death can be divided into three major groups: programmed, regulated, and accidental (Galluzzi et al., 2012). “Programmed” applies to those physiological instances of cell death that occur in the context of embryonic/post-embryonic development and tissue homeostasis (e.g., apoptosis and necroptosis). “Regulated” is used to indicate cases of cell death – be they programmed or not – whose initiation and/or execution is mediated by a dedicated molecular machinery, implying that they can be inhibited by targeted pharmacological and/or genetic interventions (e.g., autophagic death and NETosis). “Accidental” indicates cell death triggered by extremely harsh physical conditions (e.g., freeze–thawing cycles or high concentrations of pro-oxidants), which cannot be inhibited by pharmacological and/or genetic manipulation and usually exhibits morphological features of necrosis.

A form of “accidental death” is secondary necrosis, which is defined as an autolytic process of cell disintegration with release of cell components that occurs when there is no intervention of scavengers after cell death is completed (Silva, 2010). In this regard, it is important to distinguish accidental necrosis from programmed necrosis or necroptosis, a tightly controlled programmed form of death with morphological resemblance of necrosis, but that is orchestrated by the serine-threonine kinases RIP1 and RIP3, and can be specifically inhibited by necrostatins (Wu et al., 2012). Necroptosis may play a regulatory role in the development of the immune system and in the response to viral infection by serving as a backup mechanism of cell death if apoptosis is impaired (Kaiser et al., 2011; Oberst et al., 2011; Zhang et al., 2011; Mocarski et al., 2012). In principle, every form of programmed and regulated cell death may be at risk of suffering secondary necrosis if efficient clearance is delayed. Therefore, although secondary necrosis has been long considered a consequence of inappropriate clearance of apoptotic cells (Silva, 2010), it is possible that secondary necrosis may result from any form of death in which the corps is inadequately cleared.

### PROGRAMMED AND REGULATED CELL DEATH

To date, 13 modes of programmed and regulated cell death have been proposed based on biochemical pathways, the effect of specific death inhibitors, and morphological changes occurring in the dying cell and its organelles (reviewed in Galluzzi et al., 2012). The induction of each form of death depends on different factors including the cell type, the death stimuli, the cytokine environment, the presence of pathogens, and the presence of death inhibitors, among others. Thus, death by pyroptosis (a caspase-1-dependent process activated by intracellular bacteria) is restricted to macrophages and dendritic cells (DCs), ETosis (also referred as NETosis when occurs in neutrophils) appears to be specific for granulocytes, and cornification (a program of terminal differentiation that is dependent on caspase-14) is limited to keratinocytes (Galluzzi et al., 2012). Moreover, death signals can be modulated to trigger different forms of death. For example, although signals from members of the tumor necrosis factor receptor (TNFR) family activate the caspase cascade to induce death by apoptosis, inhibition of caspase-8 (e.g., by virus infection) changes the death pathway toward programmed necrosis (i.e., necroptosis; Han et al., 2011; Mocarski et al., 2012). Interestingly, lupus T cells exhibit persistent mitochondrial hyperpolarization as well as depletion of ATP and glutathione, which results in the induction of necrosis (likely necroptosis), instead of apoptosis, in response to activation-induced death (Gergely et al., 2002; Fernandez and Perl, 2009; Fernandez and Perl, 2010).

Cytokines add an additional level of complexity by modulating pathways that promote cell survival, cell death, and the mode of death. For example, in the case of neutrophils which are programmed to undergo spontaneous apoptosis as a mechanism to maintain immune system homeostasis, granulocyte-macrophage colony-stimulating factor (GM-CSF) can delay this process and prolong neutrophil survival (Geering and Simon, 2011). However, when neutrophils are exposed to GM-CSF upon ligation of CD44, the cells die by an autophagy-related form of neutrophil necroptosis (Mihalache et al., 2011). Moreover, if GM-CSF primed neutrophils are exposed to LPS or complement factor 5a (C5a), the cells generate a unique form of neutrophil extracellular traps (NETs) made of mitochondrial DNA (Yousefi et al., 2009).

In cells that may contain more than one death program, there is the potential to select specific modes of death depending on stimuli in the environment. However, once the cell is committed to die by one specific form of death, this program appears to be irreversible and in some cases incompatible with other forms of death (at least for *in vitro* studies). Thus, there are types of death that seem to be antagonistic (i.e., they cannot co-exist in the same cell) because the activation of one death pathway inhibits the others, for example, apoptosis and NETosis or apoptosis and necroptosis (Galluzzi et al., 2012). The capacity to activate diverse forms of death appears to provide an advantage to the host by switching modes of death under conditions in which specific death pathways may be inhibited, for example, in the clearance of pathogens by necroptosis when apoptosis is inhibited (Han et al., 2011; Mocarski et al., 2012). The development of highly specific probes and ability to study cell death *in vivo* will be critical to determine what form of cell death predominates in a given target tissue and may provide valuable insights into the pathogenesis of autoimmune diseases. This is particularly relevant in the study of cells that contain multiple death programs and are known to be present in areas of inflammation, such as neutrophils.

## PROGRAMMED AND REGULATED CELL DEATH IN NEUTROPHIL FUNCTION AND HOMEOSTASIS

Among the different modes of programmed and regulated cell death, at least four types have been described in neutrophils. Apoptosis and NETosis, which are known to occur *in vivo* (Yipp et al., 2012), and autophagic-like cell death and an autophagy-related form of necroptosis, which have been induced *in vitro* (von and Simon, 2007; Mihalache et al., 2011). Although autophagic cell death and programmed necrosis have been implicated in controlling both innate and adaptive immune functions (Lu and Walsh, 2012), the role of these forms of death in neutrophil function and their potential consequences in autoimmunity still need to be defined.

### NEUTROPHIL APOPTOSIS AND NETOSIS: PARTNERS IN THE FIGHT AGAINST PATHOGENS

Neutrophils are unique cells that use death as a mechanism to modulate inflammation and to ensure the efficient clearance of microorganisms during infections. Two forms of neutrophil death have been implicated in these processes, apoptosis and NETosis. Apoptosis is the mechanism by which aged neutrophils constitutively die in order to maintain homeostatic cell numbers (Cartwright et al., 1964; Geering and Simon, 2011). In addition, apoptosis plays a critical role in the innate response against bacterial, fungal, and protozoal infections (reviewed in Kennedy and DeLeo, 2009; Geering and Simon, 2011). During infection, neutrophils phagocytose bacteria, fungi and protozoa, and the ingested microorganisms are destroyed by the combination of reactive oxygen species (ROS) and antimicrobial granule components. Neutrophils contain or produce many cytotoxic molecules that can cause significant damage to surrounding tissues if the inflammatory response is not tightly regulated. The engulfment of microorganisms by neutrophils typically accelerates neutrophil apoptosis, which ultimately promotes the resolution of infection. Thus, neutrophil apoptosis guaranties the safe disposal of engulfed bacteria, leads to a loss of functional properties in neutrophils, and drives the production of anti-inflammatory cytokines through clearance of the apoptotic cells by resident or infiltrating macrophages.

Neutrophils also die by NETosis in response to pathogens. During this process, neutrophils extrude extracellular fibrillary networks composed of DNA associated with histones and granular antimicrobial proteins such as proteinase 3 (PR3), myeloperoxidase (MPO), and α-defensins, among others (Urban et al., 2009). NETs act as a mesh that traps microorganisms and facilitates their interaction with neutrophil-derived effector molecules, limiting the spread of rapidly disseminating pathogens (Brinkmann et al., 2004). In addition, NETs can induce the production of antimicrobial cytokines, such as interferon-α (IFN-α; Garcia-Romo et al., 2011; Lande et al., 2011; Villanueva et al., 2011), a relevant cytokine in the control of viral, bacterial, and protozoal infections (Bogdan et al., 2004). Thus, although neutrophil apoptosis and NETosis have been studied as separate processes that occur during infection, it is more likely that these death pathways co-exist and work cooperatively for the safe and efficient clearance of pathogens by limiting pathogen spreading, activating inflammatory antimicrobial pathways, and promoting the resolution of inflammation.

Interestingly, although neutrophils are theoretically exposed to the same environmental stimuli during infection, it is intriguing that some neutrophils become phagocytic and die by apoptosis, while others become NETotic. Indeed, in mouse neutrophils in which NETs were induced by IL-8 priming and exposure to *S. flexneri*, only a fraction (13.9 ± 1.8%) formed NETs (Li et al., 2010). Thus, it is possible that this differential response may result from distinct populations of neutrophils which are specifically programmed to activate apoptosis or NETosis in response to pathogens or cytokines. Alternatively, cytokines and/or growth factors that alter the default death program of neutrophils to prolong their survival during infection (e.g., GM-CSF) may allow neutrophils not yet committed to die by apoptosis to release NETs.

### NETs vs NETosis

Since the description of NETs, it has been controversial whether neutrophils die while extruding their intracellular material and if this phenomenon occurs *in vivo*. Initial *in vitro* studies using the non-physiological stimulus, phorbol-12-myristate-13-acetate (PMA), demonstrated that formation of NETs required rupture of the cell membrane in a process marked by increased cell permeability and exposure of inner membrane phospholipids (Fuchs et al., 2007). Although this process was clearly distinguishable from apoptosis and necrosis, the striking damage suffered by the cell supported the notion that the production of NETs was associated with neutrophil death (i.e., NETosis). In this model, NET formation required cell death, resulting in the terms NETs and NETosis being used interchangeably.

However, further studies demonstrated that when using more physiological stimuli (i.e., GM-CSF priming and subsequent short-term TLR4 ligation or C5a receptor stimulation), NETs are generated by viable cells (Yousefi et al., 2009). A recent study by Yipp et al. (2012) elegantly suggests a novel paradigm in NET formation. By directly visualizing neutrophil behavior during Gram-positive skin infections in mice and humans, it was demonstrated that viable neutrophils form NETs while crawling, resulting in widespread NET deposition in tissue. As result of this process, neutrophils were rendered anuclear, but did not lyse or exhibit features of programmed cell death. In fact, anuclear neutrophils contained bacteria, suggesting that phagolysosome maturation and NET release can be separately compartmentalized, such that bacteria cannot escape from inside the cell during NET formation. Whether the anuclear neutrophil should or should not be considered dead is questionable, and it remains unclear if these cells retain the capacity to activate other death programs. In this regard, it is important to note that similar to erythrocytes and platelets (Mason et al., 2007; Lang and Qadri, 2012), cytoplasts (anuclear neutrophils generated *in vitro*) retain full capacity to die by apoptosis (Maianski et al., 2004). In this scenario, death by apoptosis of post-NET anuclear neutrophils may guaranty the safe disposal of engulfed bacteria, their efficient clearance to avoid secondary necrosis, and the induction of anti-inflammatory cytokines by phagocytes. For practical reasons we will continue using the term NETosis as a form of death, although it may be somewhat inaccurate. Although further study is needed to understand the interplay between death programs in the neutrophil, it is clear that distinct cell death mechanisms may be active in systemic autoimmune diseases and contribute differentially to the initiation and propagation of disease.

## CLEARANCE OF DEAD CELLS AND AUTOIMMUNITY

### MECHANISMS OF DEAD CELL CLEARANCE ARE NOT UNIQUE TO APOPTOTIC CELLS

Programmed and regulated forms of cell death are physiologic processes that play critical roles in many different aspects of host development and homeostasis, including tissue turnover, proper development, and the elimination of transformed and infected cells. The rapid and efficient clearance of dead cells and debris is therefore critical to prevent the accumulation of aged, damaged, infected, or dangerous cells. Although the study of apoptosis has brought about an important understanding of pathways activated by dying cells that modulate inflammation, immune tolerance, and the efficient clearance of cell debris (reviewed in Ravichandran, 2011; Wickman et al., 2012), similar mechanisms must exist for any form of programmed or regulated cell death in order to maintain host homeostasis.

During apoptosis, dying cells advertise their presence to phagocytes through the release of soluble “find-me” signals (e.g., the lipid lysophosphatidylcholine, sphingosine 1-phosphate, CX3CL1, and the nucleotides ATP and UTP), which induce migration of phagocytes toward the dying cells. Apoptotic cells also expose “eat-me” signals on their surface (e.g., phosphatidylserine, PS) that are recognized by phagocytes through specific engulfment receptors. Interestingly, other forms of death including anoikis (death induced by detachment of anchorage-dependent cells), autophagic cell death, caspase-independent apoptosis, and necroptosis also expose PS as a mechanism for efficient non-inflammatory clearance by phagocytes (Hirt and Leist, 2003; Brouckaert et al., 2004; Petrovski et al., 2007a, 2011). In addition, cells dying by anoikis and necroptosis are efficiently engulfed by PS-independent process (Hirt et al., 2000; Petrovski et al., 2007a, 2011).

Efficient mechanisms of clearance are not limited to the recognition and engulfment of intact dead cells, but include pathways that recognize and remove necrotic cell fragments and cell debris such as DNA, histones, and RNP. These pathways have the potential to be universal in the clearance of cell debris generated independently of the mode of cell death. For example, necrotic lymphocyte debris is efficiently removed by macrophages via PS, αvβ3, CD14, CD36, and complement C1q (Bottcher et al., 2006), and debris from neutrophils dying by secondary necrosis is cleared by pathways involving thrombospondin-1 and αvβ3 (Ren et al., 2001). In both cases, cell debris is removed without eliciting inflammatory cytokine secretion. Pentraxins such as C-reactive protein (CRP), serum amyloid P component (SAP), and pentraxin 3 (PTX3) are also involved in the clearance of damaged cells and their soluble constituents. CRP binds to the membranes of damaged cells (both apoptotic and necrotic) likely via phosphatidylcholine, contributing to clearance by phagocytes (Volanakis and Wirtz, 1979; Gershov et al., 2000; Hart et al., 2005; Krysko et al., 2006). In addition, CRP binds to small nuclear RNP particles and chromatin (via histones) and is believe to be involved in the clearance of potentially autoantigenic nuclear material released from dying cells (Du Clos et al., 1988; Du Clos, 1989; Jewell et al., 1993). The avid binding of SAP to chromatin displaces H1-type histones, solubilizing chromatin fragments otherwise quite insoluble at the physiological ionic strength of extracellular fluids (Butler et al., 1990). Since SAP binds to chromatin exposed by necrotic and apoptotic cells *in vivo* (Hintner et al., 1988; Breathnach et al., 1989), it may participate in the disposal of chromatin exposed during cell death, potentially including DNA found in NETs.

Components of the complement pathway are also important in the clearance of dead cells. Early components of the component complement classical pathway bind to cells undergoing secondary necrosis, promoting their engulfment by phagocytes (Gullstrand et al., 2009). In addition, C1q binds DNA and, together with DNase I, promotes degradation of necrotic cell-derived chromatin (Gaipl et al., 2004). The complement inhibitor C4b-binding protein (C4BP) binds strongly to necrotic cells, limiting DNA release from these permeable cells and inhibits the complement cascade at the level of C3 (Trouw et al., 2005).

Chromatin released from dying cells is normally degraded by serum endonucleases, such as DNase I (Napirei et al., 2004; Nathan, 2006). In this regard, although antimicrobial peptides and C1q appear to protect NET DNA from DNase I degradation (Lande et al., 2011; Leffler et al., 2012; a process likely required to enhance NET antimicrobial activity), serum from healthy controls efficiently degrades NETs (Hakkim et al., 2010; Leffler et al., 2012), suggesting the existence of additional mechanisms of NET clearance. Certainly, further studies are necessary to define the role of known and novel clearance pathways in the removal of cells dying by newly discovered forms of cell death and determine how these processes modulate inflammation in infection and autoimmunity.

### IMPAIRED CLEARANCE OF DEAD CELLS AND AUTOIMMUNITY: INSIGHTS FROM *IN VIVO* SYSTEMS

The existence of clearance defects is critical to support the current model of cell death in autoimmunity, yet there are still important gaps in the knowledge of relevant pathways that can be modulated by therapy and the forms of death responsible for driving autoimmune diseases in humans. Complete genetic deficiencies in components involved in the clearance of dead cells are very uncommon in humans and limited to a few pathways (e.g., early components of complement and DNases; Yasutomo et al., 2001; Manderson et al., 2004; Al-Mayouf et al., 2011) but are strikingly associated with SLE, suggesting that these pathways are relevant in the protection against autoimmunity. However, while these findings have been considered strong evidence to support the role of apoptosis in autoimmunity, it is important to highlight that these pathways are also involved in the clearance of dead cells generated by other mechanisms (e.g., NETosis and necroptosis). In this regard, clearance defects cannot be used as direct evidence that apoptosis alone is responsible for triggering autoimmunity.

The study of mice with targeted disruption of specific genes associated with the clearance of dying cells (assuming that this is the only function of these genes) have been used to support the role of apoptosis in autoimmunity, but some of these models have important caveats. Interestingly, although some of these models develop lupus-like features and abnormal accumulation of apoptotic cells (e.g., C1q-, C4-, SAP-, c-Mer-, DNase I-, and MFG-E8-deficient mice; Botto et al., 1998; Bickerstaff et al., 1999; Chen et al., 2000; Napirei et al., 2000; Cohen et al., 2002; Hanayama et al., 2004), disease expression is frequently dependent on background genes (e.g., C1q-, C4-, SAP-deficient mice; Botto et al., 1998; Bickerstaff et al., 1999; Chen et al., 2000; Mitchell et al., 2002; Paul et al., 2002; Gillmore et al., 2004). In some cases, an increase in the number of apoptotic bodies, although related to the targeted gene, is not associated with disease expression (Mitchell et al., 1999). Moreover, not all clearance defects in mice lead to autoimmunity (e.g., CD14- and MBL-deficient mice) despite an abnormal *in vivo* accumulation of apoptotic material (Devitt et al., 2004; Stuart et al., 2005).

These discrepancies suggest that the development of autoimmunity is not solely dependent on the accumulation of apoptotic cells and highlight the influence of background genes on disease expression. Strikingly, it was demonstrated that the autoimmune phenotype described in some gene-targeted knock-out mice (i.e., SAP-deficient mice) might be primarily due to combinations of background genes originated from the parental 129 and C57BL/6 mouse strains (Bygrave et al., 2004; Heidari et al., 2006; Carlucci et al., 2007; widely used in the generation of gene-targeted mice, including the C1q-, C4, SAP-, DNase I-, and MFG-E8-deficient mice), which may or may not interact with the target gene. In this scenario, although a targeted gene may predispose to the accumulation of apoptotic cells (e.g., C1q-deficient mice); this may not necessarily be important or sufficient for development of the autoimmune phenotype (Mitchell et al., 2002). These nuances are important to consider when translating findings from these models to human autoimmunity and reveal important limitations in interpreting the mechanistic link between the genetic predisposition to accumulate apoptotic cells and development of disease. Further studies are necessary to exclude the possibility that other forms of death may also play a role or that other functions of these pathways not related to clearance of dead cells are not responsible for the development of autoimmunity (Carroll, 2004; Carroll and Isenman, 2012).

## DO NO HARM: A UNIVERSAL REQUIREMENT FOR PHYSIOLOGIC CELL DEATH

The end point of any physiologic form of death is to carry out a required function and be appropriately cleared, without deleterious effects to the host. However, although this process requires an anti-inflammatory ending, each form of death has distinct interactions and consequences on the immune system with pro- or anti-inflammatory effects depending on the mode of death and the circumstances associated with the induction of cell death (Petrovski et al., 2007b; Miao et al., 2010; Johansson et al., 2011; Rock et al., 2011; Han et al., 2011; Fesus et al., 2011; Remijsen et al., 2011; Schiller et al., 2012). Thus, for example, while apoptotic cell death occurring in steady-state conditions (e.g., during development and cell turnover) must be tolerogenic (Voll et al., 1997), induction of tolerance could have harmful consequences in circumstances in which apoptotic cell death is increased, but a pro-inflammatory response is required (e.g., during infections and cancer). In this scenario, there are factors (e.g., cytokines, pathogens and tumor antigens) which modulate the response to apoptotic cells, allowing the induction of adaptive immune responses if immunogenic antigens are present (Le et al., 2003; Lin et al., 2008; Alfaro et al., 2011; Rock et al., 2011; Johansson et al., 2011). Collectively, these responses help to protect the host and limit the injurious process, but can themselves cause tissue damage and disease. The precise balance between pro- and anti-inflammatory effector functions driven by dying cells and the surrounding immune players is therefore critical to determine the net effect of dying cells. When this balance is shifted, the consequences may drive an autoimmune process or allow the uncontrolled growth of transformed cells.

### ANTI-INFLAMMATORY PATHWAYS INDUCED BY DEAD CELLS ARE NOT LIMITED TO APOPTOSIS

The study of apoptosis provided initial clues that dying cells are important players in limiting inflammation. However, since no physiologic form of death should harm the host under normal circumstances, it is likely that in addition to apoptosis, other modes of death may have the capacity to provide signals to resolve inflammation and/or promote healing. After ingestion of apoptotic cells, phagocytes produce “tolerate me” cytokines (e.g., transforming growth factor β, TGF-β and IL-10), and decrease secretion of pro-inflammatory cytokines (e.g., TNFα, IL-1, and IL-12), which actively creates an anti-inflammatory milieu at sites of apoptotic cell death (Voll et al., 1997; Munoz et al., 2010). Interestingly, the efficient engulfment of necroptotic cells also inhibits the production of pro-inflammatory cytokines in macrophages (Hirt and Leist, 2003; Bottcher et al., 2006). Intriguingly, despite the view that necrotic cells are pro-inflammatory, some reports suggest that the efficient phagocytosis of these cells can also trigger anti-inflammatory and tissue repair pathways (Li et al., 2001; Ren et al., 2001; Bottcher et al., 2006). Moreover, engulfment of necrotic neutrophils by immature DCs down-regulates CD80, CD86, and CD40, rendering them unable to induce allogeneic T cell responses (Clayton et al., 2003).

Although NETosis may be viewed as a dangerous form of death based on recent associations with SLE, Wegener’s granulomatosis, and Felty’s syndrome, several antimicrobial proteins that are enriched in NETs (e.g., α-defensins and the cathelicidin LL37; Brinkmann et al., 2004; Lai and Gallo, 2009; Urban et al., 2009), also have anti-inflammatory activities which favor resolution of infection and repair of damaged tissues (reviewed in Lai and Gallo, 2009). In macrophages, α-defensins inhibit the secretion of TNFα and nitric oxide, and protect mice from a murine model of peritonitis (Miles et al., 2009). LL37 can inhibit LPS-induced cytokine release from monocytes and protects mice against endotoxin shock (Nagaoka et al., 2001). In addition, cathelicidins inhibit TLR4-mediated DC maturation and cytokine release (Di et al., 2007). LL37 also appears to play a role in skin wound healing by promoting keratinocyte migration that is required for re-epithelialization of the wound (Heilborn et al., 2003; Carretero et al., 2008). Interestingly, patients with SLE have antibodies against α-defensins (also known as human neutrophil peptides or HNPs) and LL37 (Lande et al., 2011) suggesting that autoantibodies may affect the function of these antimicrobial peptides in the context of inflammation and autoimmunity. Thus, by attenuating exacerbated inflammatory responses and stimulating certain beneficial aspects of inflammation, antimicrobial peptides in NETs may have an important role in regulating and balancing inflammatory responses. Indeed, recent evidence has shown that lupus-prone mice which are unable to generate NETs due to a deficiency in Nox-2 (phagocyte NADPH oxidase) have markedly exacerbated lupus (Campbell et al., 2012), supporting the importance of NETs in immune homeostasis.

### AUTOANTIBODIES: POTENT ADJUVANTS THAT CHANGE THE INFLAMMATORY FATE OF DEAD CELLS

Different circumstances can change the inflammatory outcome of dying cells, including pathogens, cytokines, cellular transformation, and cell damage by toxic agents, among others (Casares et al., 2005; Petrovski et al., 2007b; Miao et al., 2010; Fesus et al., 2011; Han et al., 2011; Johansson et al., 2011; Remijsen et al., 2011; Rock et al., 2011; Garg et al., 2012; Schiller et al., 2012). In 2002, Leadbetter et al. (2002) revealed an unexpected mechanism that explained how immune cells might perceive autoantigens (generated under sterile and/or non-toxic conditions) as noxious structures or DAMPs (damage-associated molecular patterns), provoking the production of pro-inflammatory cytokines. By studying mechanisms that activated transgenic rheumatoid factor (RF)-B cells, they demonstrated that IC consisting of IgG bound to mammalian chromatin effectively activated RF-B cell through a dual process involving B cell antigen receptor (BCR) recognition and delivery of the DNA to TLR9 sequestered in endosomal compartments. A few years later, the same group demonstrated that the “two-receptor” paradigm can be extended to IC containing RNA-associated autoantigens by dual BCR and TLR7 engagement (Lau et al., 2005).

Based on this model, further studies identified that although apoptotic and necrotic material has minimal inflammatory activity, autoantibodies could convert cell debris into a potent inducer of IFN-α by plasmocytoid dendritic cells (pDCs) via TLRs and FcγRIIa ligation (Bave et al., 2000, 2001, 2003; Lovgren et al., 2004). Similarly, although NETs can induce production of IFN-α by pDCs (likely a physiologic response to aid in pathogen clearance), this effect is strikingly enhanced (as much as 10-fold) by the presence of IC containing NET components and antibodies against DNA, HNP, or LL37, with anti-DNA antibodies as the most prominent amplifier of this response (Lande et al., 2011). A similar effect is likely responsible for the induction of IFN-α by pDCs in the presence of NETs and anti-RNP antibodies (Garcia-Romo et al., 2011).

The “two-receptor” paradigm has also been extended to specific autoantigens found in RA (Sokolove et al., 2011). Here, it has been demonstrated that IC containing citrullinated fibrinogen co-stimulate macrophages via the TLR4 and Fcγ receptor to induce production of TNF-α by macrophages. Although fibrinogen is not an intracellular antigen, it is possible that IC containing citrullinated autoantigens released from dying cells may have a similar inflammatory effect. Taken together, these data suggest that under normal conditions, dying cells have the capacity to modulate pro- and anti-inflammatory activities to avoid host damage. However, the presence of autoantibodies against components released by dying cells shifts this balance toward an abnormal pro-inflammatory response. Although parallel studies are necessary to determine if autoantigens released by different forms of cell death have distinct inflammatory properties, the available data suggests that autoantigens released from any source have the same capacity to form IC with autoantibodies and activate the immune system.

## NEUTROPHILS IN SYSTEMIC AUTOIMMUNE DISEASES

Since the discovery of NETs, there has been renewed interest in the neutrophil as a potential driver of autoimmune disease. The neutrophil has long been implicated in playing a variety of roles in systemic autoimmune diseases from immune effector to autoantigenic target. This stems from the ability of the neutrophil to wear many different hats during the course of an immune response from phagocyte, to secretor of cytokines, producer of anti-bacterial agents and NETs, and stimulator of adaptive immune cells. The primary role of the short-lived neutrophil in host defense is to rapidly accumulate at sights of tissue injury, in the presence or absence of infection, to protect against invasion by bacteria or fungi and then die by the mechanisms described above (Nathan, 2006).

While neutrophils are present in high numbers at the sites of autoimmune damage and are thought to play active role in disease pathogenesis, their mechanistic role in autoimmunity remains unclear. Neutrophils and leukocytoclasia (i.e., neutrophil debris) are the dominant infiltrate in vasculitis affecting small vessels in systemic autoimmune diseases (Carlson and Chen, 2006), and neutrophils are the second most abundant infiltrating cell type in dermatomyositis (DM) skin lesions (Caproni et al., 2004). In SLE, increased levels of apoptotic (Courtney et al., 1999), activated (Molad et al., 1994), and immature neutrophils (Bennett et al., 2003) are found circulating in the blood of patients, and the percentage of apoptotic and activated neutrophils positively correlates with disease activity (Courtney et al., 1999). Furthermore, neutrophils are the most abundant cell type present in RA synovial fluid and are enriched at the pannus/cartilage interface, where most tissue damage occurs (Mohr et al., 1981).

### AUTOANTIGEN EXPRESSION BY NEUTROPHILS

Interestingly, the neutrophil is also thought to be a major source of autoantigens in systemic autoimmune diseases. Neutrophil-specific autoimmunity, which is strikingly associated with the small-vessel vasculitides (i.e., microscopic polyangiitis, Wegener’s granulomatosis, Churg–Strauss syndrome, and polyarteritis nodosa), was first reported in 1982 with the identification of ANCAs in a few patients with necrotizing glomerulonephritis (Davies et al., 1982). MPO and PR3 were subsequently identified as the predominant autoantigens in 1988 and 1990, respectively (Falk and Jennette, 1988; Ludemann et al., 1990). Anti-neutrophil autoantibodies (not necessarily targeting MPO and PR3) have also been described in SS and SLE (Lamour et al., 1995; Manolova et al., 2001), with recent evidence that neutrophil antimicrobial peptides are among the antigens targeted in SLE (Lande et al., 2011). It is also important to note that neutrophils express high levels of peptidylarginine deiminase enzymes (PAD2 and PAD4; Darrah et al., 2012), which are responsible for generating citrullinated proteins, currently the most specific targets of the immune response defined in RA (Wegner et al., 2010). Thus, it appears that patients with different forms of systemic autoimmunity target ubiquitously expressed proteins as well as those uniquely expressed by neutrophils. The reason that several neutrophil-specific proteins are targeted remains unclear, but may be related to their propensity to die at sites of inflammation resulting in exposure of normally sequestered antigens to the immune system.

## MODIFIED DURING DEATH: A UNIFYING PROPERTY OF AUTOANTIGENS

Clustering and structural modification of autoantigens during apoptosis meets two key requirements for breaking tolerance to self-proteins: accessibility of the antigen to the immune system (i.e., through clustering in apoptotic blebs) and non-homeostatic state of the protein/altered self (i.e., through non-tolerized posttranslational modifications, PTMs). These features initially made apoptosis an attractive central component of the autoimmune disease paradigm (Casciola-Rosen et al., 1994; Hall et al., 2004). However, although these requirements might be highly relevant to autoimmune disease pathogenesis, they have the capacity to be met by other forms of cell death.

**FIGURE 1:**
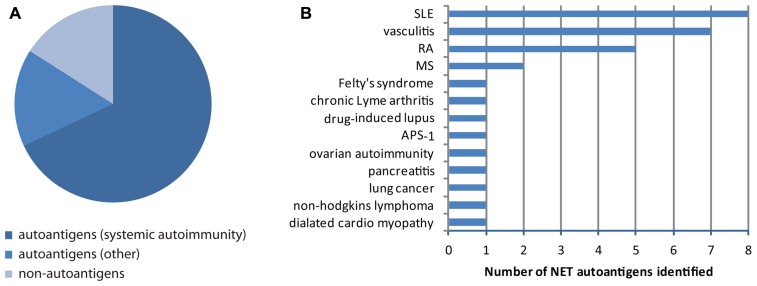
**NET autoantigens**. **(A)** Of the 25 NET components identified, 84% have been reported as autoantigens in cancer, autoimmunity, or other disorders. 74% of these proteins have been reported to be the target of autoantibodies in systemic autoimmune diseases. **(B)** The number of NET proteins reported to be autoantigens in various diseases is quantified and reveals that NET autoimmunity is most common in patients with vasculitis, SLE, and RA.

Unbiased proteomic analysis of NETs by two different groups have identified a total of 23 proteins present in these structures (Urban et al., 2009; Saffarzadeh et al., 2012), and two additional components have been described in other studies, including LL37 (Lande et al., 2011) and DNA/chromatin (Brinkmann et al., 2004; **Table 1**). These components have diverse functions and subcellular distributions in live cells, but are redistributed and extruded from neutrophils during NETosis. A literature search revealed that 84% of NET components have been identified as autoantigens in patients with autoimmunity, cancer, or both, in independent studies (**Table 1**; **Figure 1A**). In fact, 74% have been reported to be autoantigens in systemic autoimmune diseases, most dominantly in SLE, RA, and vasculitis (**Figures 1A,B**). This observation suggests that redistribution into NETs may be a previously unappreciated unifying property of several autoantigens targeted across the spectrum of autoimmune diseases and cancer.

**Table 1:** Autoantigens are enriched in NETs.

NET-protein	Autoantigen	Disease family	Disease	Reference
Actin, cytoplamsmic 1 and 2 (β/γ)*, ^ψ^	Yes	Autoimmunity	RA	Darrah et al. (2012)
α-Actinin 1 and/or 4*	Yes	Autoimmunity	SLE nephritis	Renaudineau et al. (2007)
Annexin Al^ψ^	Yes	Autoimmunity Cancer	SLE Lung cancer	Iaccarino et al. (2011) Brichory et al. (2001)
Azurocidin*	Yes	Autoimmunity	Vasculitis	Zhao et al. (1995)
Catalase*	Yes	Autoimmunity	SLE RA	Mansour et al. (2008)
Cathepsin G*	yes	Autoimmunity	Vasculitis	Schultz and Tozman (1995)
Cytokeratin-10*	Yes	Infection-induced autoimmunity	Chronic Lyme arthritis	Ghosh et al. (2006)
Neutrophil defensins (HNP)*, ^£^	yes	Autoimmunity	SLE	Lande et al. (2011)
dsDNA/chromatin^$^	Yes	Autoimmunity	SLE	Shen et al. (1998)
Leukocyte elastase*	Yes	Autoimmunity	Vasculitis	Schultz and Tozman (1995)
Leukocyte elastase inhibitor^ψ^	No			
α-Enolase*^,ψ^	Yes	Autoimmunity	RA	Wegner et al. (2010)
Glyceraldehyde 3-phosphate dehydrogenase^ψ^	Yes	Autoimmunity	Multiple sclerosis Ovarian autoimmunity SLE Dilated cardiomyopathy	Kolln et al. (2006) Edassery et al. (2010) Takasaki et al. (2004) Buse et al. (2008)
Histones*	Yes	Autoimmunity	SLE Drug-induced lupus Felty’s syndrome RA	Shen et al. (1998) Shen et al. (1998) Dwivedi et al. (2012) Sokolove et al. (2012)
Lactoferrin*^,ψ^	Yes	Autoimmunity	Autoimmune pancreatitis RA Vasculitis	Hayakawa et al. (2009) Mulder et al. (1993) Schultz and Tozman (1995)
LL37^£^	Yes	Autoimmunity	SLE	Lande et al. (2011)
Lysozyme C*	Yes	Autoimmunity	Vasculitis	Schultz and Tozman (1995)
Myeloid cell nuclear differentiation antigen*	No	–	–	–
Myeloperoxidase*^,ψ^	Yes	Autoimmunity	Vasculitis	Schultz and Tozman (1995)
Myosin-9*	Yes	Genetic autoimmunity	APS-1	Lindh et al. (2012)
Plastin-2*^,ψ^	Yes	Cancer	Non-Hodgkin’s lymphoma	Ueda et al. (2008)
Profilin-1^ψ^	No	–	–	–
Protein S100*^,ψ^	No	–	–	–
Proteinase 3*	Yes	Autoimmunity	Vasculitis	Schultz and Tozman (1995)
Transketolase*	Yes	Autoimmunity	Multiple sclerosis	Lovato et al. (2008)

* Saffarzadeh et al. (2012);

ψ Urban et al. (2009);

$ Brinkmann et al. (2004);

£ Lande et al. (2011).

*APS-1, autoimmune polyendocrine syndrome type I.*

Interestingly, although the exposure of autoantigens is a common feature shared by NETosis and apoptosis, which may similarly balance their potential relevance in autoimmunity, it is noteworthy that these processes are clearly distinguishable by the exposed autoantigens and the way that these molecules are structurally modified. Thus, in contrast to apoptotic blebs, major autoantigens in systemic autoimmune disease such as RNPs (e.g., Ro, La, Sm, and U1-70K) are not found in NETs (Urban et al., 2009; Villanueva et al., 2011; Saffarzadeh et al., 2012). Similarly, it is unknown whether neutrophils dying by NETosis expose phospholipids targeted by anti-phospholipid antibodies.

Apoptotic and NETotic cells also generate different PTMs. Depending on the stimuli, apoptotic cells can contain autoantigens modified by proteolysis (induced by caspases and/or granzymes), phosphorylation/dephosphorylation, photo-induced damage, methylation, ADP-ribosilation, and transglutamination (Casciola-Rosen et al., 1999; Rutjes et al., 1999; Hall et al., 2004; Andrade et al., 2005; Dieker et al., 2007; Utz et al., 1997, 2000; Darrah and Rosen, 2010; van Bavel et al., 2011; Bernard et al., 2012). In some cases, these modifications can be recognized by autoantibodies (e.g., phosphorylation, acetylation, and methylation) and have been suggested to play a role in the loss of tolerance to self-proteins. During NETosis, however, the induction of PTMs appears to be more limited. In this regard, methylation, citrullination, and acetylation have been detected in histones (Liu et al., 2012), but is unknown whether other autoantigens may be modified during NETosis. Moreover, although histone citrullination is a hallmark in NETs formation and a target for autoantibodies in Felty’s syndrome (Li et al., 2010; Dwivedi et al., 2012), citrullination of major autoantigens targeted in RA (e.g., enolase and vimentin; Wegner et al., 2010) have not reported to occur during NETosis. Although citrullination appears not to occur in apoptotic cells dying by camptothecin or staurosporine (Neeli et al., 2008), whether other apoptotic stimuli can activate citrullination is unknown.

Apoptosis and NETosis may therefore have distinct features (neither of which are mutually exclusive) that may offer different advantages in the disease-specific paradigms of autoimmunity. Thus, while the induction of protein citrullination, the exposure of PR3 and MPO, and the extrusion of chromatin during NETosis are attractive elements (but not exclusive for NETosis) for diseases like RA, ANCA-associated vasculitis, and SLE, respectively, features like the targeting of Ro and La and their association with photosensitivity in SLE can be more easily explained by ultraviolet-B (UVB)-induced keratinocyte apoptosis (Casciola-Rosen et al., 1994) than by NETosis. Finally, it is unlikely that apoptosis and NETosis are the only modes of death with the capacity to modify protein immunogenicity, which may add layers of complexity to the autoimmune paradigm and may support the development of disease-specific models.

## THEORICAL MODELS TO INTEGRATE NETOSIS AND APOPTOSIS INTO THE PARADIGM OF SYSTEMIC AUTOIMMUNE DISEASES

The finding that apoptotic cells and nucleosomal DNA are abnormally increased in the circulation and target tissues of patients with systemic autoimmune diseases strongly supports the notion that abnormal production and/or clearance of apoptotic cells is ongoing in this group of diseases (Yu et al., 1997; Courtney et al., 1999; Andrade et al., 2000; Makino et al., 2003; van Rossum et al., 2005; Holdenrieder et al., 2006; Lin et al., 2007; Midgley et al., 2009). In addition, apoptotic material in complex with autoantibodies can induce production of IFN-α by pDCs (Bave et al., 2000, 2001, 2003; Lovgren et al., 2004; Vollmer et al., 2005), a critical cytokine in the current paradigm of SLE (Ronnblom et al., 2011) that is gaining interest in the pathogenic model of SS, autoimmune myositis, scleroderma, and RA (Greenberg et al., 2005; Hjelmervik et al., 2005; Gottenberg et al., 2006; Walsh et al., 2007; Higgs et al., 2011, 2012; van et al., 2011). However, while it is widely accepted that apoptosis is occurring in the setting of systemic autoimmunity, it remains unclear if this process is an initiator of disease, a propagator of the feed-forward cycle of immune-mediated tissue damage, or a byproduct of unchecked inflammation.

These unanswered questions, together with the growth in our understanding of the complexities of cell death, have sparked interest in other cell death programs that may be equally or more important than apoptosis in driving autoimmunity. Recent studies on NETotic cell death have demonstrated that similar to apoptotic material, NETs (especially in complex with anti-NET antibodies) can activate IFN-α production by pDCs (Garcia-Romo et al., 2011; Lande et al., 2011; Villanueva et al., 2011), adding a novel mechanistic player into SLE pathogenesis and potentially other systemic autoimmune disease in which IFN-α may play a pathogenic role (Knight and Kaplan, 2012). Moreover, NETs have been implicated in the generation and release of autoantigens targeted in small-vessel vasculitis and Felty’s syndrome (Kessenbrock et al., 2009; Dwivedi et al., 2012). However, how NETosis may influence the role of apoptosis in the model of systemic autoimmune diseases is still unclear.

Based on the general concepts discussed in the initial part of this review and recent experimental data, we will provide four hypothetical models in an attempt to integrate apoptosis and NETosis into a pathogenic paradigm of systemic autoimmunity. It is not our intention to suggest that dying neutrophils are the only source of autoantigens or the primary target during disease initiation. Depending on the target tissue, many other cells can be the primary source of autoantigens (e.g., keratinocytes, synoviocytes, lymphocytes, monocytes, platelets, myocytes, etc.) in which case, the neutrophil may still play a role, but not as the origin of autoantigens toward which tolerance is initially broken. In addition, considering that the growing literature in regard to NETs and autoimmune diseases is biased toward SLE, these models will be largely based upon this prototypic systemic autoimmune disease. Model I will discuss the possibility that defects in common clearance pathways are the driver of systemic autoimmunity, while models II–IV suggest that apoptosis and NETosis play independent roles in systemic autoimmune disease pathogenesis. These three additional models are largely based on three independent studies in 2011, which suggested that NETosis may play a pathogenic role in SLE. While they agreed that NETs can induce IFN-α production by pDCs (with some differences about the mechanism), they differed dramatically in their conclusions about the mode by which NETs are generated (Garcia-Romo et al., 2011; Lande et al., 2011; Villanueva et al., 2011). These differences have major effects on the development of a unified pathogenic model of systemic autoimmunity.

### THEORY I: THE SHARED MECHANISM MODEL

**FIGURE 2:**
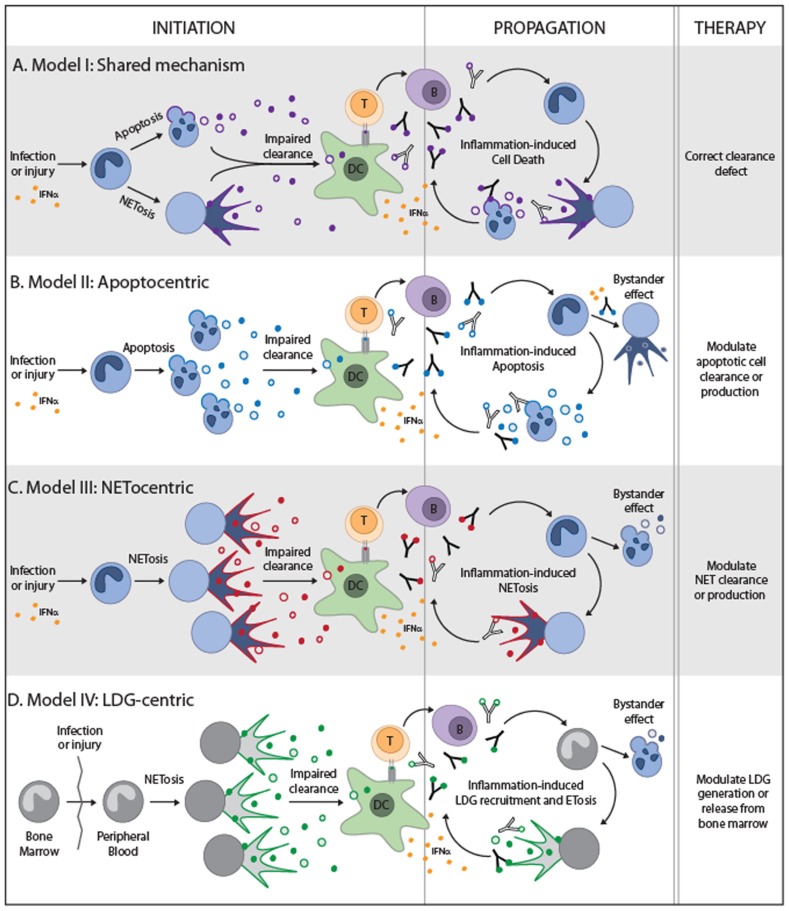
**Theoretical models to integrate NETosis and apoptosis into the paradigm of systemic autoimmune diseases**. The four models are depicted and illustrate the form of cell death that is predicted to play a role during disease initiation and propagation. For simplicity, the neutrophil is shown in all models as the initiating source of autoantigens, but other tissue sources of cell death may be the primary driver of autoimmunity in models **(A,B)**. The neutrophil and large density granulocyte (LDG) are the primary initiating sources of autoantigens in models **(C)** and **(D)**, respectively. All models require the impaired clearance of cell debris, activation of dendritic cells and production of IFNα, and presentation of autoantigens to T helper cells. T helper cells would then stimulate autoantibody production which may lead to pro-inflammatory clearance of apoptotic cells and may combine with IFNα to induce NET formation. The nuances of each model are depicted and suggested therapeutic targets.

If systemic autoimmune diseases are the result of genetic or acquired defects in universal clearance pathways, it is possible that any form of cell death may provide the signals required to activate an autoimmune response (**Figure 2A**). In this regard, it is interesting that the same clearance defects that have been used to support the role of apoptosis in autoimmune diseases are now being translated into the NETotic model of autoimmunity (e.g., impairment of DNase1 function; Martinez et al., 2008; Hakkim et al., 2010). This suggests that diseases in which abnormal accumulation or clearance of apoptotic and NETotic cells has been implicated, may result from a common defect (e.g., SLE). In this scenario, although some forms of death may dominate at distinct disease stages depending on the environment (e.g., apoptosis, necroptosis, and/or autophagic cell death during viral infections, but NETosis in bacterial or fungal infections); any form of death may have the same potential to initiate and/or to propagate the disease. Certainly, it is possible that unique autoantigen modifications (e.g., cleavage by death proteases, phosphorylation, and citrullination, among others; Hall et al., 2004; Darrah and Rosen, 2010), generated by specific forms of death, may influence autoantigen selection and disease phenotype. It is important to determine if common clearance defects are playing a dominant role in these diseases before attributing disease development to a particular death pathway since therapies targeting unique forms of death (e.g., NETosis or apoptosis) would have little effect in this setting. Instead, therapies should be focused on enhancing universal pathways involved in the anti-inflammatory clearance of dead cells.

Attempts to improve the clearance of cell debris as therapy in SLE have not been deeply explored. In 1959–1960, bovine DNase I was first used in a small number of patients with encouraging results (Lachmann, 2003). However, following clinical improvement, patients developed antibodies to the bovine DNase which precluded further treatment. Forty years later, human recombinant DNase I was used in patients with SLE in a Phase 1b trial (Davis et al., 1999). Although this study did not identify clinical benefit, it was limited by the small number of patients and by failure to achieve sufficient bioactive serum concentrations. No further studies have followed in the use of DNase or any other component involved in the clearance of dead cells for the treatment of SLE.

### THEORY II: THE “APOPTOCENTRIC” MODEL

The study by Garcia-Romo et al. (2011), demonstrated that SLE neutrophils undergo accelerated death *in vitro* (defined by trypan blue staining which indicates secondary necrosis) and identified that these cells are dying by apoptosis (determined by TUNEL assay). This data is consistent with previous observations both in pediatric and adult SLE (Courtney et al., 1999; Midgley et al., 2009), and supports the notion that in SLE, neutrophils are actively dying by apoptosis and undergoing secondary necrosis. Interestingly, the presence of anti-RNP immunoglobulin (Ig) induced a prominent reduction in the apoptosis rate of SLE neutrophils (*in vitro*) by changing the cell death program to NETosis. Moreover, although they detected a prominent type-I IFN signature in SLE neutrophils, they found that neutrophils exposed to IFN-α *in vitro* did not die by apoptosis and concluded that IFN is not responsible for the accelerated apoptosis observed in this cell type. Instead, they found that IFN-α increased the expression of TLR7 in neutrophils, which made them more susceptible to die by RNP-Ig-induced NETosis via FcγRIIa/TLR7 ligation.

Although there are some aspects here that need further mechanistic analysis (like the switch from apoptosis to NETosis), this data suggests a model in which apoptosis and NETosis co-exist in the lupus paradigm, but have different roles in the disease process (**Figure 2B**). Here, IFN-α activated neutrophils appear to be a bystander target for autoantibodies, and NETosis is a consequence of this process. In this model, the requirement of TLR-ligation suggests that cells suffering from secondary necrosis (likely from the large pool of apoptotic neutrophils in SLE) play a role in disease initiation by releasing antigens (i.e., RNP) to form IC which induce NETs. Together, this data suggest that in accordance with the apoptosis theory in SLE, accelerated apoptosis (and/or clearance defects) are likely involved in disease initiation, autoantibody production, and the early burst of IFN-α. Importantly, this model is not limited to apoptotic neutrophils, but can be applied to apoptotic material generated from any tissue in SLE. Induction of NETs by the effect of IFN-α and “two-receptor” ligation of RNP-IC may be part of an amplifying process of disease propagation that enhances the exposure of autoantigens (i.e., DNA) and endogenous adjuvants (e.g., LL37) and stimulates further IFN-α production in SLE. In this study, NETs induced by RNP-IC can activate IFN-α production by pDCs independently of FcγRIIa.

The recent finding that NETs are not necessary, but apparently protective in lupus-prone mice further support the model that NETs may be an epiphenomenon (at least in the lupus model), and open the possibility that NETs may have important effects in immunoregulation (Campbell et al., 2012). Although this finding cannot be directly translated into the human model, it certainly challenges investigators to better understand the role of NETs in inflammation in humans, before considering it as a potential target for therapy. Instead, therapies that correct the primary defect in the apoptotic pathway or enhance apoptotic cell clearance may have therapeutic benefit.

### THEORY III: THE “NETOCENTRIC” MODEL

The study by Lande et al. (2011) has novel features of interest which support a primary role for NETosis in the initiation and propagation of systemic autoimmunity (**Figure 2C**). First, they discover that patients with SLE have circulating IC containing DNA and neutrophil antimicrobial peptides (LL37 and HNPs), and that a large proportion of patients have autoantibodies against LL37 and HNP. Second, LL37 and HNP are expressed on the surface of SLE neutrophils and this process is likely induced by IFN-α. Third, mouse monoclonals against LL37 and HNP induced NETs in SLE neutrophils and in IFN-α primed neutrophils. Finally, the study showed that freshly isolated SLE neutrophils release DNA in culture and suggested that this process reflects the spontaneous production of NETs by SLE patient neutrophils. Although this study neither addressed whether circulating IC containing DNA-LL37/HNPs are indeed generated from NETs (but not other mode of neutrophil death), whether human anti-LL37 and anti-HNP antibodies can trigger NET formation, nor confirmed that the DNA released spontaneously by SLE neutrophils was from NETs, the data suggests a model in which NET production in SLE results from a vicious cycle whereby autoantibodies target NET components and induce further NET formation by IFN-α activated neutrophils. In this study, although NETs can induce IFN-α production by pDCs, this process is strongly increased by IC formation with anti-DNA, anti-LL37, or anti-HNP antibodies.

This data supports a more “NETocentric” model that the previous one (Theory II) because the critical autoantigens that may be sufficient to initiate disease (i.e., DNA, LL37, and HNP) can be directly exposed in NETs. Thus, in the context of an adequate genetic predisposition, the abnormal clearance of NETs generated during infection may predispose individuals to the production of anti-DNA, anti-LL37, and anti-HNP antibodies. The presence of these autoantibodies in combination with subsequent infections may amplify an interferogenic response until compensatory mechanisms are surpassed, leading to disease propagation and development of clinical symptoms. In this model, apoptosis may occur as result of chronic inflammation and tissue damage induced by NETs (likely providing additional autoantigens not expressed in neutrophils), and suggests that the finding of accelerated apoptosis in SLE may correspond to an epiphenomenon not necessarily associated with the induction of disease. In contrast to the previous models, therapies to improve NET clearance or decrease NET production may be useful to ameliorate disease.

### THEORY IV: THE LOW-DENSITY GRANULOCYTE-CENTRIC MODEL

The last model of NETs in systemic autoimmunity is based on the study by Villanueva et al. (2011); **Figure 2D**. The major finding in this study is strikingly different from the others (Garcia-Romo et al., 2011; Lande et al., 2011); the authors conclude that that low-density granulocytes (LDGs), but not mature neutrophils, are responsible for generating NETs in SLE. LDGs appear to represent an immature form of granulocyte that is prematurely released from the bone marrow in patients with SLE and RA (Hacbarth and Kajdacsy-Balla, 1986; Denny et al., 2010). Because their morphological features are quite different from mature neutrophils, LDGs are co-purified with mononuclear cells during peripheral blood cell gradient separation using Ficoll-Hypaque. Contamination with LDGs appears to be responsible for the prominent granulocytic signature found in peripheral mononuclear cells (PBMCs) from patients with SLE (Bennett et al., 2003; Denny et al., 2010). Strikingly, Villanueva et al. (2011) found that LDGs spontaneously generate NETs immediately after purification. However, in contrast to the data by Lande et al. (2011), mature SLE neutrophils showed no differences in spontaneous NET formation compared to controls.

There are some noteworthy features about LDGs that make them unique as the source of NETs in SLE. Immature neutrophils do not respond to type I IFN (e.g., IFN-α) because they fail to phosphorylate STAT1 (signal transducer and activator of transcription 1) in response to receptor binding (Martinelli et al., 2004), a feature likely shared by LDGs. This finding is consistent with gene expression analysis of lupus LDGs in which among 302 differentially expressed genes (compared to control neutrophils) there is no evidence of IFN-induced gene activation (Villanueva et al., 2011). Thus, this may indirectly support the hypothesis that IFN-α plays no role in the activation or generation of LDGs. Moreover, in contrast to other studies in which the pre-existence of IFN-α is required to prime neutrophils to generate NETs (Garcia-Romo et al., 2011; Lande et al., 2011), this study suggests that IFN-α may have no role in inducing NETs by LDGs, providing a model in which LDGs may precede IFN-α production in SLE. Instead, this study shows that IL-17 is exposed in LDG-NETs, suggesting that this cytokine may be involved in tissue damage and immune dysregulation induced by LDG-NETs. An intriguing part of this study is that although SLE neutrophils do not generate NETs spontaneously; these cells in culture can release DNA (the mechanism is undefined) that can induce IFN-α mRNA expression by a pDC cell line in a similar proportion to LDG-NETs. In this study, the effect of anti-NET antibodies on IFN-α production by pDCs was not addressed.

It is still unknown whether circulating LDGs are found before clinical disease initiation in SLE and the mechanism(s) by which these cells are activated and abnormally released from bone marrow. However, it is possible that the abnormal release and NETosis of LDGs (a process potentially boosted during infection) may be the initial trigger for the aberrant production of IFN-α in SLE. Moreover, abnormally cleared NETotic LDGs may serve as source of autoantigens for autoantibody production in SLE. In this model, the abnormal apoptotic cell death found in SLE would be a consequence of the autoimmune process and may contribute to disease propagation, but is not the primary cause of disease. Importantly, this model offers a unique and specific target (LDGs) of therapy for the treatment of SLE.

## CONCLUSION

The discovery of NETs has brought renewed interest in the neutrophil as a dominant player in the pathogenesis of systemic autoimmune diseases. It is clear that NET formation by neutrophils is a biological phenomenon with pathogenic potential, but how this relates to apoptosis in the pathogenic model of systemic autoimmune diseases remains undefined. This review puts forth several models to integrate the growing body of data on NETosis with the historically appreciated role of apoptosis in these diseases. The study of NETs is still in its infancy and in order to adopt a new pathogenic model, further studies are necessary to determine how these structures and neutrophil remains (i.e., post-NET anuclear neutrophils) modulate the immune response. Moreover, the pathogenic mechanisms and pathways by which NETs are generated need to be identified and will require the development of highly specific probes to study these structures *in vivo*. Although NETs can be distinguished morphologically when induced *in vitro*, the study of NETs in tissues requires specific markers to clearly distinguish NETosis from other types of granulocyte damage that may release DNA and cytoplasmic contents (e.g., secondary necrosis). In this regard, measuring soluble dsDNA (e.g., in plasma or in supernatants; Margraf et al., 2008; Lande et al., 2011) to quantify NETosis is questionable, since other forms of death (e.g., apoptosis and necrosis) may also be responsible for this effect (van der Vaart and Pretorius, 2008), especially in diseases with a high rate of apoptosis and secondary necrosis, such as SLE (Garcia-Romo et al., 2011).

Undoubtedly, the early findings about the potential pro-inflammatory effect of NETs are stimulating and have rejuvenated the study of cell death in systemic autoimmune diseases. As more forms of cell death are discovered and the growing complexity of cell death is understood, it is important to reexamine the role of different forms of cell death in the generation and modification of autoantigens. This will require the discovery of useful markers to distinguish unique forms of death in blood and tissues and may have important implications in the development of novel therapies that target cell death pathways in the treatment of systemic autoimmune diseases.

## Conflict of Interest Statement

The authors declare that the research was conducted in the absence of any commercial or financial relationships that could be construed as a potential conflict of interest.
